# A critical appraisal of the quality of adult musculoskeletal ultrasound guidelines using the AGREE II tool: an EuroAIM initiative

**DOI:** 10.1007/s13244-017-0563-4

**Published:** 2017-07-28

**Authors:** Carmelo Messina, Bianca Bignotti, Alberto Tagliafico, Davide Orlandi, Angelo Corazza, Francesco Sardanelli, Luca Maria Sconfienza

**Affiliations:** 1 Scuola di Specializzazione in Radiodiagnostica, Università degli Studi di Milano, Via Festa del Perdono 7, 20122 Milan, Italy; 2 Department of Health Sciences, University of Genova, Genoa, Italy; 3 University of Genova and AOU IRCCS San Martino IST, Genoa, Italy; 4 Ospedale Evangelico Internazionale, Piazzale Gianasso 5, 16100 Genoa, Italy; 5 Unità Operativa di Radiologia Diagnostica ed Interventistica, IRCCS Istituto Ortopedico Galeazzi, Via Riccardo Galeazzi 4, 20161 Milan, Italy; 6 Servizio di Radiologia, IRCCS Policlinico San Donato, Piazza Malan 1, 20097 San Donato Milanese, Italy; 7 Dipartimento di Scienze Biomediche per la Salute, Università degli Studi di Milano, Via Pascal 36, 20133 Milan, Italy

**Keywords:** Musculoskeletal, Ultrasonography, Guidelines, AGREE, Evidence-based medicine

## Abstract

**Objectives:**

Our aim was to evaluate the quality of published guidelines on musculoskeletal ultrasound (MSK-US) for adults.

**Methods:**

Between June and July 2016, we conducted an online search for MSK-US guidelines, which were evaluated by four independent readers blinded to each other using the AGREE II tool. A fifth independent reviewer calculated scores per each domain and agreement between reviewers’ scores using the intraclass correlation coefficient (ICC).

**Results:**

Five guidelines were included in this appraisal. They were published between 2001 and 2014. Our appraisal showed intermediate results, with four out of five guidelines scoring “average” as overall quality. Domain 1 (scope and purpose) achieved the highest result (total score = 71.1% ± 18.7%). Domain 6 (editorial independence) had the lowest score (total score = 26.3% ± 19.3%). Interobserver agreement was very good for all the evaluated guidelines (ICC ranged between 0.932 and 0.956).

**Conclusions:**

Overall, quality of MSK-US guidelines ranges from low to average when evaluated using the AGREE II tool. The editorial independence domain was the most critical, thus deserving more attention when developing future guidelines.

***Main messages*:**

*• Four of five guidelines on MSK-US had an average quality level.*

*• Scope/purpose had the highest score (71.1% ± 18.7%).*

*• Editorial independence had the lowest score (26.3% ± 19.3%).*

*• Interobserver agreement was very good (ranges: 0.932–0.956).*

**Electronic supplementary material:**

The online version of this article (doi:10.1007/s13244-017-0563-4) contains supplementary material, which is available to authorised users.

## Introduction

Ultrasound (US) is currently considered the preferred first-line imaging modality for investigating several musculoskeletal disorders [1, 2]. Recent improvements in US technology has resulted in an increased diagnostic accuracy for a wide spectrum of musculoskeletal diseases affecting tendons, ligaments, muscles, and nerves both at upper and lower limbs [1, 3–7]. Further advantages of US are, among others, its non-invasiveness, being in particular a radiation-free modality, as well as the wide availability of US systems and low cost [8, 9]. For these reasons, the use of musculoskeletal US (MSK-US) is rapidly increasing among physicians worldwide [1].

At the same time, the widespread use of MSK-US has raised the need for “evidence-based” recommendations to help physicians in choosing when US is more indicated in the daily routine and how to perform a technically correct US examination. In fact, appropriate use of US examinations is fundamental for increasing the diagnostic accuracy as well as to reduce the number of unnecessary examinations [1, 10]. For these reasons, some guidelines have been developed in recent years with recommendations that include indications for MSK-US examination, which anatomical part or structure to evaluate, or the most relevant clinical conditions for which MSK-US is indicated [1, 11–14]. Such guidelines are usually developed by medical bodies or specialised working groups and play a key role in clinical practice and are a reference in the case of malpractice case litigation [10], as they should provide indications based on the highest level of evidence. This evidence is usually achieved by critical selection and evaluation of systematically searched original studies and meta-analyses [15, 16]. However, according to the methodology of guideline development, variation in quality may occur. Thus, it is crucial to evaluate the methods on which a guideline is developed [17, 18].

Different quality appraisal tools have been developed for the evaluation of guidelines. Among them, the Appraisal of Guidelines for Research & Evaluation version II (AGREE II) has been reported to be a reliable, internationally used, and validated tool [19].

The European Network for the Assessment of Imaging in Medicine (EuroAIM), a joint initiative of European Institute for Biomedical Imaging Research, was initiated with the aim to increase the evidence for the rational use of medical imaging [15, 20]. Recently, EuroAIM has been focusing its activity on the evaluation of guidelines in different fields of diagnostic imaging [21]. For what concerns musculoskeletal radiology, a conjoined project between EuroAIM and the European Society of Musculoskeletal Radiology (ESSR) was established [21], and MSK-US was included among the topic of interests.

The aim of this study was to evaluate the quality of current guidelines on MSK-US for adults using the AGREE II quality assessment tool.

## Materials and methods

Between June and July 2016, we performed a literature search for MSK-US guidelines using PubMed, EMBASE, Google, and the Wiley Online Library, with the following keywords: “musculoskeletal ultrasound”, “musculoskeletal ultrasonography”, “Guidelines”, “Indications”, “Official Positions” and their expansions. Once guidelines were retrieved, their references were screened for further papers to include. We excluded from the results of our search those papers that were not primarily focused on MSK-US, such as guidelines about imaging of specific disorders in which US was described in the setting of a more comprehensive evaluation. Inclusion criteria were as follows: full manuscript available in English; guidelines issued by national or international medical bodies; guidelines mainly focused on the evaluation of musculoskeletal disorders by means of US; guidelines might contain recommendation on the MSK-US anatomy technique; guidelines must focus mainly on the adult population (age > 18 years).

Guideline quality evaluation was made using the AGREE II instrument via the official website dedicated online platform [19]. The AGREE II protocol consist of 23 different items organised in 6 domains: domain 1 = “Scope and Purpose” (items 1–3); domain 2 = “Stakeholder Involvement” (items 4–6); domain 3 = “Rigor of Development” (items 7–14); domain 4 = “Clarity of Presentation” (items 15–17); domain 5 = “Applicability” (items 18–21); domain 6 = “Editorial Independence”. These six domains are followed by two additional items (“Overall Assessment”), which includes “the rating of the overall quality of the guideline and whether the guideline would be recommended for use in practice”. Table 1 shows a detailed description of all AGREE II items [19].

**Table 1:** Summary of AGREE II structure and detailed list of items within each domain (from reference 15)

**Domain 1. Scope and purpose**	
Item 1	The overall objective(s) of the guideline is (are) specifically described
Item 2	The health question(s) covered by the guideline is (are) specifically described
Item 3	The population (patients, public, etc.) to whom the guideline is meant to apply is specifically described.
**Domain 2: Stakeholder involvement**	
Item 4	The guideline development group includes individuals from all the relevant professional groups.
Item 5	The views and preferences of the target population (patients, public, etc.) have been sought.
Item 6	The target users of the guideline are clearly defined.
**Domain 3: Rigor of development**	
Item 7	Systematic methods were used to search for evidence.
Item 8	The criteria for selecting the evidence are clearly described.
Item 9	The strengths and limitations of the body of evidence are clearly described.
Item 10	The methods for formulating the recommendations are clearly described.
Item 11	The health benefits, side effects, and risks have been considered in formulating the recommendations.
Item 12	There is an explicit link between the recommendations and the supporting evidence.
Item 13	The guideline has been externally reviewed by experts prior to its publication.
Item 14	A procedure for updating the guideline is provided.
**Domain 4: Clarity of presentation**	
Item 15	The recommendations are specific and unambiguous.
Item 16	The different options for management of the condition or health issue are clearly presented.
Item 17	Key recommendations are easily identifiable.
**Domain 5: Applicability**	
Item 18	The guideline describes facilitators and barriers to its application.
Item 19	The guideline provides advice and/or tools on how the recommendations can be put into practice.
Item 20	The potential resource implications of applying the recommendations have been considered.
Item 21	The guideline presents monitoring and/or auditing criteria.
**Domain 6: Editorial independence**	
Item 22	The views of the funding body have not influenced the content of the guideline.
Item 23	Competing interests of guideline development group members have been recorded and addressed.

Four independent reviewers (CM, BB, DO, AC) with 4 to 14 years’ experience in MSK-US and scientific research gave a score to each guideline. Reviewers were selected by the EuroAIM working group among the members of the US subcommittee of the ESSR. All reviewers were previously trained to use the AGREE II scoring system through the user manual, which is available on the online platform; in addition, reviewers were asked to complete two online training tools specifically developed to assist users in effectively applying the instrument. According to the instruction tool, each item was rated on a 7-point scale, which ranges from 1 (strongly disagree, which means that no relevant information is provided) to 7 (strongly agree, which means that the quality of reporting is exceptional). Final domain scores were calculated by summing up scores of all items within the domain and by scaling the total as a percentage of the maximum possible score for that domain [available via http://www.agreetrust.org/about-the-agree-enterprise/introduction-to-agree-ii/scoring-the-agree-ii/].

### Data analysis

For analysis purposes, the evaluations performed by each reviewer were averaged; the average of each domain is reported in the results. Agreement between reviewers’ scores was calculated using the intraclass correlation coefficient (ICC), defined as follows: <0.20, poor; 0.21–0.40, fair; 0.41–0.60, moderate; 0.61–0.80, good; 0.81–1.00, very good. As for previous studies, the overall quality of each guidelines was evaluated using a threshold of 60% for the final score of each domain [22, 23]. High quality was defined when 5 or more domains scored >60%, average quality when 3 or 4 domains scored >60%, low quality when ≤2 domains scored >60%. In addition, the total score (expressed as mean ± standard deviation, SD) of guidelines and domains was calculated. Domain scores were categorised as good (≥ 80%), acceptable (60–79,9%), low (40–59,9%), or very low (<40%), similar to a previous similar paper [24]. Data extraction, collection, and scoring were done by a fifth independent reviewer (LMS) with 14 years of experience in musculoskeletal US and scientific research, using a Microsoft Excel® 2016 spreadsheet. ICC calculations were performed using the SPSS software (version 24, IBM, Armonk, NY, USA).

## Results

Our strategy of literature search identified 206 papers, 5 of which met our inclusion criteria [1, 11–14]. The characteristics of MSK-US guidelines that were included in our study are presented in Table 2. Year of publication ranged from 2001 to 2014.

**Table 2:** General characteristics of MSK-US guidelines included in the analysis

MSK-US guideline title	Country of origin	Year of publication	Organization
Musculoskeletal ultrasound: technical guidelines [11]	European Union	2010	European Society of Musculoskeletal Radiology (ESSR)
Clinical indications for musculoskeletal ultrasound: a Delphi-based consensus paper of the European Society of Musculoskeletal Radiology [1]	European Union	2012	European Society of Musculoskeletal Radiology (ESSR)
Guidelines for musculoskeletal ultrasound in rheumatology [14]	European Union	2001	European League Against Rheumatism (EULAR) Working Group for MSK-US
AIUM Practice Parameter for the Performance of a MSK US Examination [12]	USA	2012	American Institute of Ultrasound in Medicine (AIUM)
ACR–AIUM–SPR–SRU Practice Parameter for the Performance of the Musculoskeletal Ultrasound Examination [13]	USA	2014	American College of Radiology (ACR), American Institute of Ultrasound in Medicine (AIUM), Society for Pediatric Radiology (SPR), Society of Radiologists in Ultrasound (SRU)

Table 3 summarises the total score for each domain as well as the final judgment of overall quality. Detailed scores and reviewers’ comments for each guideline are reported in Supplementary Tables 1 to 5. Four out of five guidelines reached an *average* level of quality, having 3 or 4 domains with a score higher than 60%. Among these, “Clinical indications for musculoskeletal ultrasound” by Klauser et al. [1] on behalf of the ESSR achieved the highest total score (66.2%). Only the “Guidelines for musculoskeletal ultrasound in rheumatology” by Backaus et al. [14] on behalf of EULAR had a *low* level of quality, with only two domains scoring >60% and a total score of 35.9% ± 20.6%. The paper “Musculoskeletal ultrasound: technical guidelines” by Martinoli [11] on behalf of the ESSR was the guideline with the greatest score variability, with a SD of 29.7%, while the rheumatologic guideline issued by EULAR [14] had the lowest variability (SD = 20.6%).

**Table 3:** Summary of the average of domain scores of MSK-US guidelines according to AGREE II

Guideline Title	Domain 1	Domain 2	Domain 3	Domain 4	Domain 5	Domain 6	Total score mean (SD)	Overall quality
Musculoskeletal ultrasound: technical guidelines [11]	73.6%* (acceptable)	62.5%* (acceptable)	9.9% (very low)	83.3% * (good)	30.2% (very low)	25.0% (very low)	47.4% (29.7%)	Average
Clinical indications for musculoskeletal ultrasound: a Delphi-based consensus paper of the European Society of Musculoskeletal Radiology [1]	87.5% * (good)	58.3% (low)	74.5% * (acceptable)	87.5% * (good)	31.3% (very low)	58.3% (low)	66.2% (21.5%)	Average
Guidelines for musculoskeletal ultrasound in rheumatology [14]	38.9% (very low)	41.7% (very low)	14.6% * (very low)	61.1% * (acceptable)	51.0% (low)	8.3% (very low)	35.9% (20.6%)	Low
AIUM Practice Parameter for the Performance of a MSK US Examination [12]	76.4% * (acceptable)	63.9% * (acceptable)	30.2% (very low)	70.8%* (acceptable)	55.2% (low)	14.6% (very low)	51.9% (24.4%)	Average
ACR–AIUM–SPR-SRU Practice Parameter for the Performance of the Musculoskeletal Ultrasound Examination [13]	79.2% * (acceptable)	61.1% * (acceptable)	34.4% (very low)	66.7% * (acceptable)	63.5% * (acceptable)	25.0% (very low)	55.0% (20.8%)	Average
**Total domain score; mean (SD)**	**71.1% (18.7%) (acceptable)**	**57.5% (9.1%) (low)**	**32.7% (25.5%) (very low)**	**73.9% (11.2%) (acceptable)**	**46.3% (14.9%) (low)**	**26.3% (19.3%) (very low)**		

Domain 1 = scope and purpose; domain 2 = stakeholder involvement; domain 3 = rigor of development; domain 4 = clarity of presentation; domain 5 = applicability; domain 6 = editorial independence. Domain scores ≥80% = good; 60–79% = acceptable; 40–59% = low; <40% = very low. * = total score of domain >60%. High quality was defined when 5 or more domains scored >60%, average quality when 3 or 4 domains scored >60%, low quality when ≤2 domains scored >60%. AIUM = American Institute of Ultrasound in Medicine; ACR = American College of Radiology; SPR = Society for Pediatric Radiology; SSR = Society of Skeletal Radiology; SD = standard deviation

Domain scores ranged between 8.3% (lowest value, domain 6 of EULAR Guidelines [14]) and 87.5% (highest value, domain 1 of ESSR “Clinical Indications for Musculoskeletal Ultrasound” [1]). When comparing the scores of each domain across guidelines, “Clarity of Presentations” (domain 4) and “Scope and Purpose” (domain 1) achieved the highest results, with a total domain score of 73.9% ± 11.2% and 71.1% ± 18.7%, respectively. The domain with the lowest total score was “Editorial Independence” (domain 6), with a total mean score of 26.3% ± 19.3%.

Total mean score of domain 1 (“Scope and Purpose”) was 71.1% with moderate variability (SD = 18.7%). The ESSR “Clinical Indications for Musculoskeletal Ultrasound” [1] reached the highest score (87.5% = good), while the EULAR guidelines [14] had a “very low” score of 38.9%.

Total mean score of domain 2 (“Stakeholder Involvement”) was 57.5% with the lowest variability (SD = 9.1%). Within this domain, AIUM guidelines [12] had the highest score (63.9% = acceptable), while the EULAR guidelines [14] had the lowest score (41.7% = very low).

Domain 3 (“Rigor of Development”) was the one with the second lowest mean score (32.7%) and, overall, the higher variability (SD = 25.5%). This variability is related to the considerable difference between the guideline with the highest domain score (ESSR “Clinical Indication for Musculoskeletal Ultrasound” [1], total score of 74.5%) and the technical guideline by Martinoli [11], of which the final domain score was 9.9% (“very low”).

For domain 4 (“Clarity of Presentation”), the overall mean score was “acceptable” with a mean score of 73.9%. Quality scores variability was 3.6% SD. The ESSR “Clinical indications for musculoskeletal ultrasound” [1] was the guideline with the highest score (87.5% = good), while the EULAR guidelines [14] scored the lowest value (61.1% = acceptable).

Domain 5 (“Applicability”) had a mean quality score of 46.3% with moderate variability (SD = 9.3%). Guideline scores varied from the maximum value of 63.5% (acceptable) from the ACR–AIUM–SPR–SRU [13] conjoined guideline to the lowest value of 30.2% (very low) of the technical guideline by Martinoli [11].

Overall, the domain with the lowest score was domain 6 (“Editorial Independence”), with a mean score of 26.3%. This domain had the second larger variability, with a SD of almost 20%. As for the other domains, this variability is influenced by the difference between the highest and lowest domain score. The ESSR “Clinical indications for musculoskeletal ultrasound” [1] had the highest value of 58.3%, still considered “low”. Conversely, EULAR guidelines [14] reached the score of 8.3% (very low), which was the lowest value among all domains.

Interobserver variability ranges were 0.956 (very good; 95% confidence interval, 0.917–0.979) for the ESSR “Musculoskeletal ultrasound: technical guidelines” [11]; 0.932 (very good; −0.872-0.968) for ESSR “Clinical indications for musculoskeletal ultrasound” [1]; 0.936 (very good; −0.879-0.970) for EULAR guidelines [14]; 0.944 (very good; −0.895-0.974) for AIUM guidelines [12]; and 0.941 (very good, −0.889-0.972) for the ACR–AIUM–SPR–SRU conjoined guidelines [13].

## Discussion

Our main finding is that the AGREE II appraisal of MSK-US guidelines showed intermediate results, with an overall quality that was “average” in four out of five guidelines and many domains that scored “low” or “very low”. In addition, even though a high variability was found across the six domains, a “good” domain score (i.e. a percentage higher than 80%) was reached only three times. When considering the within-domain scores, variability was somehow smaller, with domain 2 (stakeholder involvement) showing the lowest SD.

One of the reasons for these results may be that four guidelines [11–14] are mainly technical guidelines, dealing mostly or only with the practical execution of MSK-US examinations (e.g., how to orient the probe, position the patient, visualise a tendon). Therefore, their purpose is to provide technical education rather than literature evidence on the topic. In this setting, not all domains evaluated by the AGREE II tool may be relevant to this kind of recommendation. Nevertheless, AGREE II developers clearly state that this tool “can be applied to guidelines in any disease area targeting any step in the health care continuum”, therefore opening the possibility to technical guidelines appraisal as well. Of note, the ESSR “Clinical indications for musculoskeletal ultrasound” [1] is a guideline based on a Delphi process which occurred after a thorough review of pertinent literature and was aimed to provide clinical indications about the degree of evidence of the use of MSK-US in different parts of the body. This may explain why this guideline was the one with the highest overall score. However, excluding technical guidelines, we acknowledge a relative low number of clinical guidelines on MSK-US.

Domains with the highest quality were “clarity of presentation” (domain 4) and “scope and purpose” (domain 1), as both scored over 70%. Although less evident in our appraisal, this is comparable to previous papers that evaluated other guideline with the AGREE II tool, regardless of the topic [23–26]. The reason of such high specific domain scores is still unclear [23]. A possible explanation may be the fact that domains 1 and 4 contain fundamental guideline parts that cannot be easily omitted, such as the objectives, the health question being addressed, and the population to whom the guideline is applied. Thus, guideline developers probably pay more attention to these parts when developing their papers.

Editorial independence (domain 6) scored “very low” in all guidelines, except for the paper ESSR “Clinical indications for musculoskeletal ultrasound”, whose final score was “low”. Therefore, this was the domain with the poorest score (26.3%). A similar situation was reported by Armstrong et al. in a recent appraisal of osteoporosis guidelines, with domain 6 presenting with the lowest score (45%) [23]. The score of this domain was low in several other studies [24, 25, 27, 28], with few exceptions [26]. Regarding “Editorial Independence” evaluation, the AGREE II considers two items related to funding bodies or potential authors’ competing interests that may have influenced the guideline content [19]. To accomplish AGREE II criteria, an explicit statement that the funding body interests have not influenced the final recommendations should be present; at the same time, all guideline authors should provide a disclosure of all competing interests. According to reviewers, this information is not adequately reported in all guidelines, especially for the EULAR paper, which obtained a score lower than 10%. This aspect remains critical, as it has been shown that conflicts of interest among authors of guidelines are very common and may affect the quality of final recommendations [23, 29–31]. Therefore, particular attention to the quality of this domain is obviously needed, especially for those guidelines containing recommendation on diagnostic technologies or medications.

According to the AGREE II instrument, a substantial increase in the overall quality score of MSK-US was observed from 2001 to 2012, but not so from 2012 to 2014. Compared to EULAR guidelines [14] published in 2001, the paper by ESSR [1] published in 2012 had an improvement in the final total score of about 30%. Our results differ from a 2012 review by Kung et al. [32] who found that very small improvement of medical guidelines occurred over the previous two decades. On the other side, a paper published in 2016 by Armstrong et al. shows similar values of quality improvement over time [23]. However, the number of guidelines evaluated is relatively low, so that more time is necessary to confirm this trend.

Importantly, interobserver agreement was very good for all the evaluated guidelines; hence, all reviewers were quite unanimous about the conferred scores. In fact, all reviewers’ comments were somehow uniform, a finding that further confirm the reviewers’ confidence with the AGREE II appraisal system.

This study has limitations, some of them being intrinsic to the AGREE II system. In fact, this tool is not aimed at evaluating all aspects of a guideline. First, AGREE II does not evaluate the degree of consistency between guideline recommendation and the reported evidence [24]. Also, AGREE II does not evaluate the scientific and clinical content, a limitation that is common to several appraisal tools [33]. Finally, the four reviewers of this appraisal have different experiences in MSK-US and guidelines evaluation, potentially biasing the outcome. However, the use of average scores and previous training on the proper of AGREE II instrument should have reduced the impact of this limitation.

In conclusion, our study showed that overall quality of MSK-US guidelines is intermediate according to the AGREE II evaluation. Domain 6 (Editorial Independence) was the most critical considering the overall score; therefore, emphasis should be given to these aspects, to produce recommendations with a lower risk of bias.

## Electronic supplementary material

ESM 1 (DOCX 20 kb)

ESM 2 (DOCX 20 kb)

ESM 3 (DOCX 20 kb)

ESM 4 (DOCX 20 kb)

ESM 5 (DOCX 20 kb)

## References

[1] Klauser AS, Tagliafico A, Allen GM (2012). Clinical indications for musculoskeletal ultrasound: a Delphi-based consensus paper of the European Society of Musculoskeletal Radiology. Eur Radiol.

[2] Lesniak BP, Loveland D, Jose J (2014). Use of ultrasonography as a diagnostic and therapeutic tool in sports medicine. Arthrosc J Arthrosc Relat Surg.

[3] Corazza A, Orlandi D, Fabbro E (2015). Dynamic high-resolution ultrasound of the shoulder: how we do it. Eur J Radiol.

[4] Tagliafico AS (2016). Peripheral nerve imaging: not only cross-sectional area. World J Radiol.

[5] Tagliafico A, Bignotti B (2015). New parameters for evaluating peripheral nerve disorders on sonography and magnetic resonance imaging. J Ultrasound Med.

[6] Filippou G, Adinolfi A, Iagnocco A (2016). Ultrasound in the diagnosis of calcium pyrophosphate dihydrate deposition disease. A systematic literature review and a meta-analysis. Osteoarthr Cartil.

[7] Sconfienza LM, Orlandi D, Lacelli F (2015). Dynamic high-resolution US of ankle and midfoot ligaments: normal anatomic structure and imaging technique. Radiographics.

[8] Derchi L, Rizzato G, Bianchi S, Martinoli C (2007). Technical Requirements. Ultrasound musculoskeletal system.

[9] Messina C, Banfi G, Aliprandi A (2016). Ultrasound guidance to perform intra-articular injection of gadolinium-based contrast material for magnetic resonance arthrography as an alternative to fluoroscopy: the time is now. Eur Radiol.

[10] Zappia M, Aliprandi A, Pozza S (2016). How is shoulder ultrasound done in Italy? A survey of clinical practice. Skelet Radiol.

[11] Martinoli C (2010). Musculoskeletal ultrasound: technical guidelines. Insight Imaging.

[12] American Institute of Ultrasound in Medicine (2012) AIUM Practice Parameter for the Performance of a Musculoskeletal Ultrasound Examination. http://www.aium.org/resources/guidelines/musculoskeletal.pdf. Accessed 1 Apr 2017

[13] ACR–AIUM–SPR–SRU (2014) Practice Parameter for the Performance of the Musculoskeletal Ultrasound Examination. https://www.acr.org/~/media/ACR/Documents/PGTS/guidelines/US_Musculoskeletal.pdf

[14] Backhaus M, Burmester GR, Gerber T (2001). Guidelines for musculoskeletal ultrasound in rheumatology. Ann Rheum Dis.

[15] Sardanelli F, Bashir H, Berzaczy D (2014). The role of imaging specialists as authors of systematic reviews on diagnostic and interventional imaging and its impact on scientific quality: report from the EuroAIM evidence-based radiology working group. Radiology.

[16] Egger M, Smith GD, Altman DG (2001) Systematic reviews in health care: meta-analysis in context. BMJ Books

[17] Shaneyfelt TM, Mayo-Smith MF, Rothwangl J (1999). Are guidelines following guidelines? The methodological quality of clinical practice guidelines in the peer-reviewed medical literature. JAMA.

[18] Grilli R, Magrini N, Penna A (2000). Practice guidelines developed by specialty societies: the need for a critical appraisal. Lancet (Lond, Engl).

[19] Brouwers MC, Kho ME, Browman GP (2010). AGREE II: advancing guideline development, reporting and evaluation in health care. CMAJ.

[20] EIBIR European Network for the Assessment of Imaging in Medicine. http://www.eibir.org/scientific-activities/joint-initiatives/euroaim/. Accessed 27 Dec 2016

[21] Messina C, Bignotti B, Bazzocchi A et al (2017) A critical appraisal of the quality of adult dual-energy X-ray absorptiometry guidelines in osteoporosis using the AGREE II tool: an EuroAIM initiative. Insights Imaging. doi:10.1007/s13244-017-0553-6 10.1007/s13244-017-0553-6 PMC5438319 28432574

[22] Ou Y, Goldberg I, Migdal C, Lee PP (2011). A critical appraisal and comparison of the quality and recommendations of glaucoma clinical practice guidelines. Ophthalmology.

[23] Armstrong JJ, Rodrigues IB, Wasiuta T, MacDermid JC (2016). Quality assessment of osteoporosis clinical practice guidelines for physical activity and safe movement: an AGREE II appraisal. Arch Osteoporos.

[24] Sekercioglu N, Al-Khalifah R, Ewusie JE (2017). A critical appraisal of chronic kidney disease mineral and bone disorders clinical practice guidelines using the AGREE II instrument. Int Urol Nephrol.

[25] Vasse E, Vernooij-Dassen M, Cantegreil I (2012). Guidelines for psychosocial interventions in dementia care: a European survey and comparison. Int J Geriatr Psychiatry.

[26] Zeng L, Zhang L, Hu Z (2014). Systematic review of evidence-based guidelines on medication therapy for upper respiratory tract infection in children with AGREE instrument. PLoS One.

[27] Holmer HK, Ogden LA, Burda BU, Norris SL (2013). Quality of clinical practice guidelines for glycemic control in type 2 diabetes mellitus. PLoS One.

[28] Zhang Z, Guo J, Su G (2014). Evaluation of the quality of guidelines for myasthenia gravis with the AGREE II instrument. PLoS One.

[29] Norris SL, Holmer HK, Ogden LA, Burda BU (2011). Conflict of interest in clinical practice guideline development: a systematic review. PLoS One.

[30] Norris SL, Holmer HK, Ogden LA (2012). Conflict of interest disclosures for clinical practice guidelines in the National Guideline Clearinghouse. PLoS One.

[31] Neuman J, Korenstein D, Ross JS, Keyhani S (2011). Prevalence of financial conflicts of interest among panel members producing clinical practice guidelines in Canada and United States: cross sectional study. BMJ.

[32] Kung J, Miller RR, Mackowiak PA (2012). Failure of clinical practice guidelines to meet institute of medicine standards: two more decades of little, if any, progress. Arch Intern Med.

[33] Vlayen J, Aertgeerts B, Hannes K (2005). A systematic review of appraisal tools for clinical practice guidelines: multiple similarities and one common deficit. Int J Qual Health Care.

