# Supplementary material for: A critical appraisal of the quality of adult musculoskeletal ultrasound guidelines using the AGREE II tool: an EuroAIM initiative
Source: Insights Imaging. 2017 Jul 28;8(5):491–7. doi: 10.1007/s13244-017-0563-4 (PMC5621989; doi:10.1007/s13244-017-0563-4)
Supplement: Supplementary file 2 — (DOCX 20 kb) [file 13244_2017_563_MOESM2_ESM.docx]

Supplementary Table 2 Detailed AGREE II domain scores for the guideline “Clinical indications for musculoskeletal ultrasound” [1]

| **Domain** | **Item** | **Rater 1** | **Rater 2** | **Rater 3** | **Rater 4** | **Total** | **Total per Domain** | **Domain score** |
| --- | --- | --- | --- | --- | --- | --- | --- | --- |
| Scope and Purpose | ***1*** | 6 | 7 | 7 | 6 | 26 | 75 | **87,5%** |
|  | ***2*** | 5 | 7 | 7 | 6 | 25 |  |  |
|  | ***3*** | 5 | 6 | 7 | 6 | 24 |  |  |
| Stakeholder Involvement | ***4*** | 5 | 7 | 6 | 6 | 24 | 54 | **58,3%** |
|  | ***5*** | 3 | 2 | 1 | 2 | 8 |  |  |
|  | ***6*** | 5 | 4 | 7 | 6 | 22 |  |  |
| Rigour of Development | ***7*** | 7 | 7 | 7 | 7 | 28 | 175 | **74,5%** |
|  | ***8*** | 7 | 7 | 7 | 7 | 28 |  |  |
|  | ***9*** | 4 | 7 | 5 | 6 | 22 |  |  |
|  | ***10*** | 7 | 7 | 7 | 7 | 28 |  |  |
|  | ***11*** | 5 | 6 | 7 | 6 | 24 |  |  |
|  | ***12*** | 6 | 7 | 7 | 7 | 27 |  |  |
|  | ***13*** | 4 | 4 | 1 | 2 | 11 |  |  |
|  | ***14*** | 4 | 1 | 1 | 1 | 7 |  |  |
| Clarity of Presentation | ***15*** | 7 | 6 | 7 | 6 | 26 | 75 | **87,5%** |
|  | ***16*** | 5 | 4 | 7 | 6 | 22 |  |  |
|  | ***17*** | 6 | 7 | 7 | 7 | 27 |  |  |
| Applicability | ***18*** | 5 | 4 | 3 | 2 | 14 | 46 | **31,3%** |
|  | ***19*** | 4 | 6 | 4 | 4 | 18 |  |  |
|  | ***20*** | 1 | 1 | 1 | 1 | 4 |  |  |
|  | ***21*** | 4 | 3 | 1 | 2 | 10 |  |  |
| Editorial Independence | ***22*** | 7 | 3 | 6 | 6 | 22 | 36 | **58,3%** |
|  | ***23*** | 4 | 2 | 4 | 4 | 14 |  |  |
