# Supplementary material for: A critical appraisal of the quality of adult musculoskeletal ultrasound guidelines using the AGREE II tool: an EuroAIM initiative
Source: Insights Imaging. 2017 Jul 28;8(5):491–7. doi: 10.1007/s13244-017-0563-4 (PMC5621989; doi:10.1007/s13244-017-0563-4)
Supplement: Supplementary file 3 — (DOCX 20 kb) [file 13244_2017_563_MOESM3_ESM.docx]

Supplementary Table 3 Detailed AGREE II domain scores for the guideline “Guidelines for musculoskeletal ultrasound in rheumatology” [14]

| **Domain** | **Item** | **Rater 1** | **Rater 2** | **Rater 3** | **Rater 4** | **Total** | **Total per Domain** | **Domain score** |
| --- | --- | --- | --- | --- | --- | --- | --- | --- |
| Scope and Purpose | ***1*** | 2 | 3 | 2 | 3 | 10 | 40 | **38,9%** |
|  | ***2*** | 3 | 4 | 3 | 4 | 14 |  |  |
|  | ***3*** | 3 | 5 | 4 | 4 | 16 |  |  |
| Stakeholder Involvement | ***4*** | 4 | 5 | 4 | 5 | 18 | 42 | **41,7%** |
|  | ***5*** | 2 | 3 | 2 | 3 | 10 |  |  |
|  | ***6*** | 5 | 2 | 4 | 3 | 14 |  |  |
| Rigour of Development | ***7*** | 2 | 1 | 2 | 1 | 6 | 60 | **14,6%** |
|  | ***8*** | 2 | 1 | 2 | 1 | 6 |  |  |
|  | ***9*** | 2 | 1 | 2 | 1 | 6 |  |  |
|  | ***10*** | 2 | 1 | 2 | 1 | 6 |  |  |
|  | ***11*** | 3 | 3 | 3 | 3 | 12 |  |  |
|  | ***12*** | 1 | 2 | 2 | 1 | 6 |  |  |
|  | ***13*** | 3 | 2 | 3 | 2 | 10 |  |  |
|  | ***14*** | 3 | 1 | 2 | 2 | 8 |  |  |
| Clarity of Presentation | ***15*** | 5 | 5 | 5 | 5 | 20 | 56 | **61,1%** |
|  | ***16*** | 5 | 2 | 4 | 3 | 14 |  |  |
|  | ***17*** | 5 | 6 | 5 | 6 | 22 |  |  |
| Applicability | ***18*** | 4 | 6 | 4 | 4 | 18 | 65 | **51,0%** |
|  | ***19*** | 5 | 6 | 5 | 5 | 21 |  |  |
|  | ***20*** | 4 | 3 | 4 | 3 | 14 |  |  |
|  | ***21*** | 3 | 3 | 3 | 3 | 12 |  |  |
| Editorial Independence | ***22*** | 2 | 1 | 1 | 2 | 6 | 12 | **8,3%** |
|  | ***23*** | 2 | 1 | 2 | 1 | 6 |  |  |
